# A Rare Case of Ileal Intussusception Caused by Primary Small Bowel Trichobezoar and Meckel's Diverticulum in an Autistic Child

**DOI:** 10.1002/ccr3.70745

**Published:** 2025-08-04

**Authors:** Marwa Messaoud, Habib Ullah Joya, Amani N. Alansari, Abir Jouini, Amine Ksiaa, Ahmed Zrig, Lasaad Sahnoun

**Affiliations:** ^1^ Pediatric Surgery Department, Fattouma Bourguiba Hospital University of Monastir Monastir Tunisia; ^2^ Department of Pediatric Surgery Hamad Medical Cooperation Doha Qatar; ^3^ Radiology Department, Fattouma Bourguiba Hospital University of Monastir Monastir Tunisia

**Keywords:** autism, bowel obstruction, intussusception, Meckel's diverticulum, primary small bowel trichobezoars

## Abstract

Intussusception is a condition in which one part of the intestine slides into an adjacent part of the intestine. Intussusception is an important cause of an acute abdomen and the second most common cause of bowel obstruction in children. Trichobezoars, which are rare in children and often linked to psychiatric disorders, seldom cause intestinal intussusception. While Rapunzel syndrome—a form of gastric trichobezoar extending into the small bowel—is a recognized cause, primary small‐bowel trichobezoars are exceptionally rare. We report a unique pediatric case of ileo‐ileal intussusception triggered by a 30‐cm primary small‐bowel trichobezoar coexisting with Meckel's diverticulum, an association not previously documented. A 6‐year‐old autistic boy presented with symptoms suggestive of bowel obstruction. Imaging suggested small‐bowel intussusception related to Meckel's diverticulum. Surgery revealed an ileo‐ileal intussusception secondary to a 30‐cm obstructive trichobezoar located proximal to the invagination and an inflamed Meckel's diverticulum. The diverticulum was resected, the trichobezoar was removed, and ileo‐ileal anastomosis was performed, with no postoperative complications. The combination of a primary small‐bowel trichobezoar and Meckel's diverticulum leading to intussusception is exceedingly rare and poses unique diagnostic and therapeutic challenges, particularly in special pediatric populations. Early recognition and surgical intervention are essential to prevent bowel ischemia, perforation, and sepsis.

## Graphical abstract

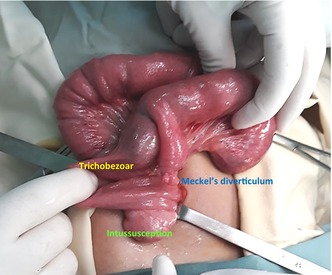

**Summary**

- Pediatric intestinal intussusception, often idiopathic, may rarely stem from lead points like Meckel's diverticulum or primary small bowel trichobezoars.
- Diagnosis is challenging in autistic/psychiatric cases due to communication barriers.
- Early recognition and surgery prevent life‐threatening complications (e.g., ischemia, perforation), emphasizing evaluation of rare etiologies in special pediatric populations.

## 1 Introduction

Intussusception is a common cause of acute abdominal emergencies in infants and children. More than 90% of the cases of intussusception in children are idiopathic, but about 5% have a pathological lead point, such as from lymphoid hyperplasia, Meckel diverticulum, duplication cyst, intestinal polyps, mesenteric nodes, lymphoma, and rarely trichobezoars that initiate the problem [1].

Trichobezoars, which are rare gastrointestinal masses formed by ingested hair that resists digestion, predominantly occur in the stomach of children and adolescents [2]. Over time, entangled hair mixes with food and mucus, potentially migrating to the small bowel and causing complications such as obstruction, intussusception, or perforation [2, 3]. While strongly linked to psychiatric disorders like trichotillomania and trichophagia [4], their association with autism spectrum disorder (ASD) remains uncommon [5]. Meckel's diverticulum is a congenital remnant of the omphalomesenteric duct with an incidence of 2% in children [6]. It can present as abdominal pain, bleeding, intussusception, intestinal obstruction, perforation, fistulas, or umbilical sinuses. Trichobezoars and Meckel's diverticulum both can independently trigger intussusception [2, 6]. The coexistence of these conditions adds complexity to diagnosis and management, often necessitating specialized treatment approaches (5). We report a unique case of a 6‐year‐old autistic boy presented with ileal intussusception caused by a primary small bowel trichobezoar and Meckel's diverticulum. To our knowledge, this specific combination has not been previously reported.

## 2 Case Report

### 2.1 Case History and Examination

A 6‐year‐old male with a known diagnosis of autism spectrum disorder (ASD). No significant surgical history. He was referred to the pediatric emergency department in February 2020 due to abdominal pain, bilious vomiting, and abdominal distension. Notably, there were no accompanying symptoms, such as fever, changes in appetite, or weight prior to admission. Parents denied any hair‐pulling behavior or hair loss.

Upon examination, the patient was afebrile, exhibited stable vital signs, and had no alopecia. The abdominal examination revealed distension without any palpable masses. A rectal examination could not be performed due to the child's uncooperative behavior.

### 2.2 Differential Diagnosis

Appendicitis, Intussusception, complicated Meckel's diverticulum, intestinal malrotation/volvulus.

### 2.3 Investigations

**FIGURE 1:**
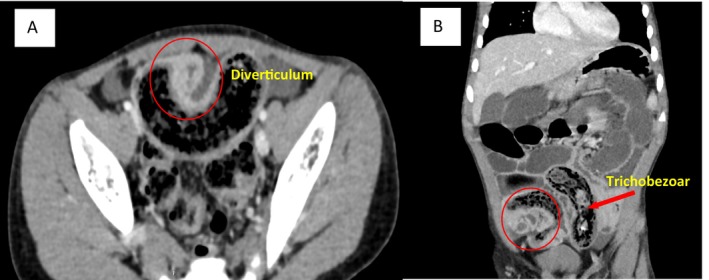
Computed tomography (CT) axial (A) and coronal (B) images demonstrating small‐bowel obstruction with dense matter accumulation in the terminal ileum (red arrow), and additional findings suggesting a complicated Meckel's diverticulum (red circle).

Initial labs showed microcytic hypochromic anemia (hemoglobin: 9 g/dL). Other parameters were within normal limits. An abdominal X‐ray revealed multiple air‐fluid levels and distension of the stomach and small bowel, prompting further evaluation with a computed tomography (CT) scan. The CT imaging indicated an acute mechanical obstruction of the small bowel, characterized by multifocal ileal wall thickening suggestive of inflammatory involvement and dense matter accumulation in the terminal ileum. Additional findings raised the possibility of a complicated diverticulum Figure 1.

## 3 Surgical Procedure

A McBurney incision was chosen over a midline laparotomy based on preoperative imaging that localized the pathology to the distal ileum, consistent with Meckel's diverticulum, typically found within 80 cm of the ileocecal valve. The absence of gastric or extensive trichobezoars on imaging further justified this limited approach, which provided adequate visualization and access without requiring incision extension.

**FIGURE 2:**
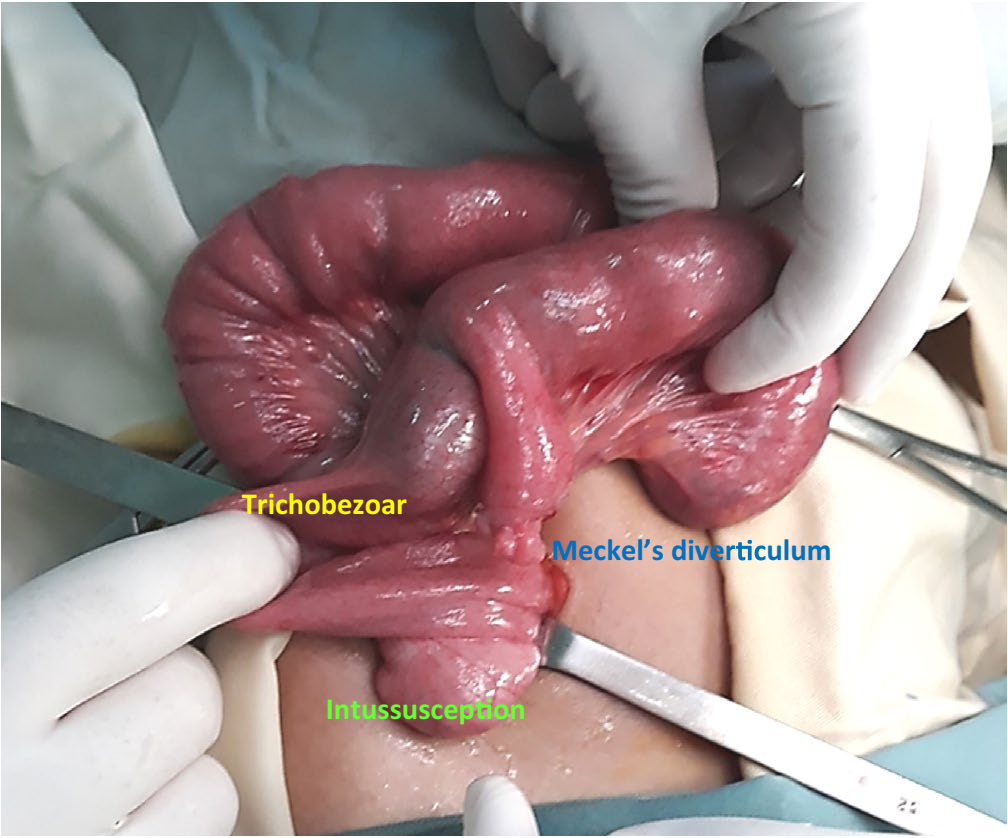
Intussusception located at the distal ileum, downstream from an obstructive trichobezoar and a Meckel diverticulum.

**FIGURE 3:**
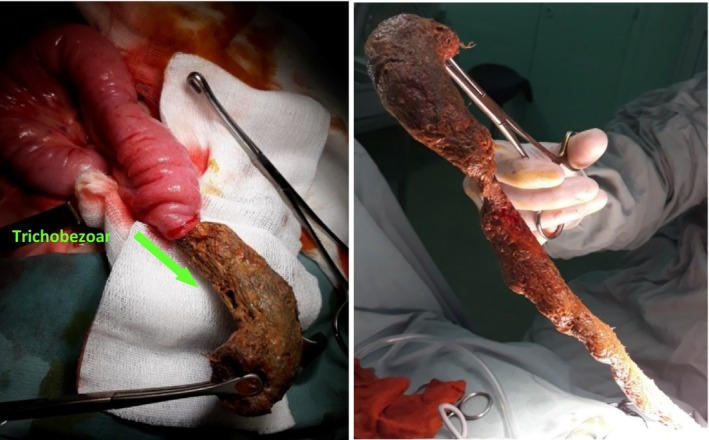
Intraoperative view showing the enterotomy and extraction of a 30 cm trichobezoar via retrograde manipulation.

Intraoperative findings revealed an intussusception located in the distal ileum, downstream from an obstructive trichobezoar measuring approximately 35 cm in length, as well as a Meckel's diverticulum situated approximately 30 cm from the ileocecal valve Figure 2. The surgical intervention involved an enterotomy for the retrograde externalization of the trichobezoar, resection of 1 cm of ileal tissue on either side of the base of the Meckel's diverticulum, terminal ileo‐ileal anastomosis, and appendectomy Figure 3. No blood transfusion was required during surgery as the blood loss was minimal. Histological examination indicated an intestinal segment containing a trichobezoar, accompanied by acute ulcerative inflammation of its wall. Meckel's diverticulum had intestinal‐type mucosa and acute inflammation, without any ectopic tissue such as gastric or pancreatic heterotopia.

## 4 Postoperative Course

The patient recovered uneventfully and was discharged on postoperative Day 7 with oral ferrous sulfate for borderline anemia. He was referred and is still under psychiatry follow‐up for ASD‐related issues. Over 4 years of regular follow‐up, no recurrence of trichobezoar or gastrointestinal symptoms has been observed.

## 5 Discussion

Gastrointestinal symptoms (GIS) are highly prevalent in children with autism spectrum disorder (ASD), arising from multifactorial mechanisms such as gut dysbiosis, immune dysregulation, food sensitivities (e.g., gluten/casein), and sensory processing differences [7, 8]. Common manifestations include abdominal pain, bloating, chronic constipation, diarrhea, encopresis, and gastroesophageal reflux disease [7, 8]. These symptoms, along with communication barriers in ASD, often delay diagnosis and treatment of acute abdominal conditions, increasing risks of complications [4].

Trichobezoar‐induced intussusception typically results from Rapunzel syndrome, which is a rare medical condition in which hair ingestion (trichophagia) occurs, usually as a manifestation of an underlying psychiatric disorder [9]. Rapunzel syndrome, characterized by a gastric trichobezoar extending into the bowel, predominantly affects females [10]. The shorter hair length in males may reduce the risk, as ingested hairs are more likely to pass into the small intestine. Unlike Rapunzel syndrome, primary intestinal bezoars lack a gastric anchoring mass and are less likely to cause intussusception [9, 10].

Patients with Autism Spectrum Disorder (ASD) can present diagnostic challenges due to communication barriers, potentially leading to complications and increased mortality risk if not adequately assessed [11].

Physical examination is crucial for detecting abdominal distress in vulnerable pediatric populations, especially those exhibiting atypical eating behaviors. In this case, the patient presented with bilious vomiting, a hallmark sign of intestinal obstruction. The diagnostic assessment was further complicated by the absence of fever or notable changes in appetite prior to admission. On examination, abdominal distension was observed without palpable masses.

In patients with autism spectrum disorder (ASD), communication barriers may limit the reliability of clinical examination for diagnosis. Nonspecific symptoms such as abdominal pain, vomiting, and distension can resemble various intestinal pathologies, complicating clinical assessment. Therefore, imaging plays a vital role in confirming intestinal obstruction and detecting associated anomalies or complications.

Abdominal X‐ray is the initial imaging modality for assessing intestinal obstruction, identifying key signs such as air‐fluid levels, gastric and small bowel distension, intraluminal mottled gas patterns, and potential perforation [12]. Although plain radiographs may suggest the presence of a trichobezoar, they lack specificity in determining its location and extent. Ultrasonography, a crucial first‐line imaging tool in pediatric gastrointestinal evaluation, typically visualizes trichobezoars as echogenic masses and assesses abdominal free fluid and potential intussusception [4]. In this case, ultrasound was not performed due to significant digestive distension, necessitating advanced imaging for further evaluation.

Computed tomography (CT) is highly effective for characterizing gastrointestinal pathologies in children. CT scans delineate trichobezoar size and configuration [12]. In our patient, CT revealed multiple air‐fluid levels and localized terminal ileum thickening, confirming an obstructive trichobezoar and incidentally identifying an inflamed Meckel's diverticulum. Early imaging aids surgical planning and reduces complications from delayed diagnosis.

The interplay of trichobezoar, intussusception, and Meckel's diverticulum is noteworthy. Intestinal intussusception occurs when a segment of bowel telescopes into an adjacent portion, rarely caused by a pathological lead point such as a trichobezoar. In this case, the Meckel's diverticulum, along with the trichobezoar, likely contributed to intussusception due to heightened peristaltic activity triggered by attempts to propel the trichobezoar through the ileocecal valve into the colon.

Treatment options for trichobezoars depend on size and location [2]. Surgical exploration remains the gold standard for large trichobezoars, enabling thorough gastrointestinal examination and management of complications (e.g., satellite trichobezoars or intestinal damage) [2, 4]. In our case, based on CT findings, a McBurney incision was chosen as the surgical approach, providing sufficient access for the successful removal of the trichobezoar and excision of Meckel's diverticulum.

Laparoscopic surgery, though minimally invasive, is typically reserved for smaller masses due to potential risks such as prolonged operative time and the possibility of spillage of the bezoar contents [2]. Endoscopic removal may be considered for small gastric or duodenal trichobezoars [13].

Given the association with autism spectrum disorder (ASD), comprehensive psychiatric care, including evaluations, cognitive‐behavioral therapy, pharmacotherapy, and close monitoring—is critical to address psychological factors and prevent recurrence [14, 15].

## 6 Conclusion

This report highlights a unique triad of primary ileal trichobezoar, intussusception, and Meckel's diverticulum in an autistic child, underscoring the need for high clinical suspicion in ASD patients with GIS. Multidisciplinary care—combining prompt imaging, surgical intervention, and psychiatric support—is essential to address complex presentations and improve outcomes in those patients.

## Author Contributions

**Marwa Messaoud:** conceptualization, data curation, investigation, methodology, writing – original draft, writing – review and editing. **Habib Ullah Joya:** conceptualization, methodology, writing – original draft, writing – review and editing. **Amani N. Alansari:** conceptualization, methodology, supervision, writing – review and editing. **Abir Jouini:** data curation, investigation. **Amine Ksiaa:** investigation, methodology. **Ahmed Zrig:** investigation, methodology. **Lasaad Sahnoun:** investigation, methodology.

## Ethics Statement

The Patient's parents were informed and agreed that data concerning the case would be submitted for publication. The Medical Research Fattouma Bourguiba Hospital, University of Monastir, Monastir, Tunisia confirmed the patient's consent, confirmed that data was anonymized, and agreed with publication.

## Consent

Written informed consent was obtained from the parents for the publication of this case report and the accompanying images.

## Conflicts of Interest

The authors declare no conflicts of interest.

## Data Availability

Data will be made available on request.
